# The 0–2 Transition of CO in Condensed Oxygen, Nitrogen, and Argon

**DOI:** 10.6028/jres.068A.032

**Published:** 1964-06-01

**Authors:** Stanley Abramowitz, H. P. Broida

## Abstract

Infrared absorption spectra of CO in the region of the first overtone have been observed in dilute (approximately 1 to 10 parts in 1000) liquid solutions of oxygen, nitrogen, and argon, and clear crystalline nitrogen and argon matrices. The overtone band was found at 4249.0, 4252.4, and 4252.0 cm^−1^ with half widths of 18.4, 17.8, and 13.7 cm^−1^ in liquid oxygen, nitrogen, and argon solutions at 82, 78, and 82 °K, respectively. The half width in liquid oxygen varied from 18.4 to 10.0 cm^−1^ in the temperature range 82 to 57 °K. The band position was the same but its width was smaller in the crystalline nitrogen matrix. Two bands were observed in the clear crystalline argon solid at 4245 and 4256 cm^−1^. The solution results cannot be interpreted with the recent theory of Buckingham.

Infrared absorption spectra of carbon monoxide in the region of the first overtone have been observed in the liquid solvents oxygen, nitrogen, and argon. In addition, the spectra have been obtained in clear crystalline solutions of argon and of nitrogen near the triple points of these solvents. The purpose of these experiments was to determine the influence of temperature, phase changes, and solvents on half width, position, and shape of the CO absorption band.

A Perkin-Elmer model 99 monochromator with a 2000 lines/cm grating blazed at 10° (1.7*μ* in first order) was used in the first order with a spectral slit width of about 1 cm^−1^. An antireflection coated germanium filter eliminated the higher orders from the 1000 w tungsten filament lamp used as the light source. The quartz absorption cell used, recently described by Bass and Broida [1], was modified slightly by recessing the windows further into the coolant tube. The resultant increased thermal contact between the refrigerant and the solution greatly simplified the growing of the clear crystalline matrices. The temperature of the refrigerant, liquid oxygen, was regulated by pumping on it with a small vacuum pump (capacity 14 liters/min). The vapor pressure of the liquid oxygen refrigerant, measured with an aneroid type gauge, provided an indication of the temperature. The direct measurement of the vapor pressure above the solution with a mercury manometer also provided an indication of the temperature. Solutions were prepared from the gases which had been mixed in the ratios of 1 to 10 parts carbon monoxide to 1000 parts of the various solvents. The position, the half width, and the shape of the spectral band did not depend on the concentration in this range. The clear solid solutions were grown slowly from the liquids at or near the triple points of the solvents.

The measured frequencies and half widths of the 0–2 transition of CO in condensed oxygen, nitrogen, and argon are summarized in table 1. In liquid oxygen and liquid nitrogen, the band was symmetrical both at the half width and twice the half width and had a Lorentzian shape (using the width at twice the half-band width as the criterion [2]). There were no changes in the position or the shape of the band in liquid oxygen at temperatures from 57 to 82 °K. However the half-band width varied from 10.0 to 18.4 cm^−1^ in this temperature range. In liquid argon the band is slightly asymmetric with more absorption on the high-frequency side.

Although the position of the band in clear crystalline solid nitrogen is not greatly different from that of the corresponding liquid solution, the half width is reduced by one-third and the shape is asymmetric and broader on the high-frequency side in the solid matrix. The absorption in the wings of the band is less than one would expect for a Lorentzian band shape. This observation is in apparent agreement with Wieder and Dows [3] who recently have observed vibrational bands of solid C_2_H_4_ and C_2_D_4_ which had shapes between the Gaussian and Lorentzian forms. In clear crystalline argon, the band is split into two overlapping peaks with the high-frequency peak about 50 percent more intense than the other peak.

Results for the band positions obtained in this study are in good agreement with the recently published results of Vu, Atwood, and Vodar [4]. The band contours which are shown by them appear quite similar to the ones observed in this study but half widths were not listed, so that a further comparison of our results with theirs is not possible. These workers did not study the influence of temperature on the spectrum.

In an effort to find an explanation for the observations of the 0–2 band of CO in condensed phases, several theoretical models have been tried. Unfortunately none of these theories easily account for the band shapes and shifts.

Ewing [5] has observed the CO fundamental vibration in the liquid phase, in nitrogen and argon solutions. The bands he observed were not only asymmetric but also broader than the 0–2 bands observed in this study. The carbon monoxide fundamental had half widths of 26 cm^−1^ and 18 cm^−1^ in liquid nitrogen and argon, respectively, at temperatures comparable to those in this study. Ewing ascribed the asymmetry and increased absorption to the high-frequency side of the bands to a low barrier to rotation. From the asymmetry he estimated the barrier to be 42 cm^–1^ in pure liquid carbon monoxide, while slightly lower and slightly higher barriers were estimated for carbon monoxide solutions in liquid nitrogen and argon, respectively. A comparison with the present results (table 1) shows that the harmonic band is about two-thirds as broad as that of the fundamental. Moreover, the lack of asymmetry of the band in liquid nitrogen coupled with its narrowness in comparison to Ewing’s observations of the fundamental seems to indicate that either hindered rotation is not the disordering mechanism contributing to the band width observed, or the barrier is several times higher than *kT.*

The observation of a variation of the half width of the CO harmonic in liquid oxygen from 10.0 to 18.4 cm^−1^ in the temperature range of 57 to 82 °K is surprising in the light of existing theories. If the band contour is determined principally by a strong collision process, in which the duration of the collision is small compared to the time between collisions, a Lorentzian band contour should be observed. The half width of such a band should be proportional to *T*^1/2^ [6, 7]. The observed dependence is clearly a function of a higher power of the temperature.

If hindered rotation is responsible for the band width, then an increase in half-band width and asymmetry to the high-frequency side of the band is to be expected with a rise in temperature if the barrier is comparable to *kT.* If the barrier is much higher than *kT* the band width is independent of temperature. Since the population of the *J* levels of a rotator is proportional to *T*^1/2^, one would expect the width of the band to vary roughly as *T*^1/2^ if free or hindered rotation is causing the observed breadth. The observed dependence of approximately *T*^3/2^ coupled with the lack of asymmetry seems to rule out this explanation for CO in oxygen.

It has recently been suggested by Rakov in application to organic materials that the width of bands could be represented by an exponential of the form

$$\Delta v_{1/2}=A\;\exp\;\left(-E/RT\right)$$ (1)

where *E* is the potential barrier for reorientation of the molecules [8, 9]. Rakov lias further indicated that if Brownian motion is responsible for the observed band widths this *E* should be equivalent to the energy of viscous flow, *E*_vis_, which is defined by Glasstone, Laidler, and Eyring [10] through the relationship

$$\eta =B\;\exp\;\left(E_{v\;\text{is}}/RT\right)$$ (2)

where *η* is the viscosity of the liquid medium. Using the data in table 1 for the band width of the CO harmonic in liquid oxygen at 57 °K and 82 °K, one may calculate the *E* appearing in eq (1). This value of *E* is about a factor of two smaller than the *E*_vis_ calculated for liquid oxygen in this temperature range from the available data on the viscosity of liquid oxygen [11]. It appears therefore that this theory does not fit the phenomena observed in this study.

Recently Buckingham [12, 13, 14] presented a theory to account for solvent effects on vibrational transitions of diatomic molecules. One of the unique predictions of this theory is that the (*s—*1) overtone of a diatomic molecule should be *s* times as broad as the fundamental. The half widths observed in this study of the first overtone of carbon monoxide are decidedly *smaller* than the widths of the fundamental in these same solvent systems observed by Ewing [5]. This indicates the failure of Buckingham’s theory in predicting band widths for the simple system carbon monoxide in nitrogen and argon solutions. The solvent shifts (*v*_vap_ —*v*_sol’n_) observed for the carbon monoxide harmonic in nitrogen is 7.6 cm^−1^, which is about 2.5 times the solvent shift of 3 cm^−1^ for the fundamental observed by Ewing. Buckingham’s theory as well as the earlier theory of Kirkwood, Bauer, and Magat [15, 16] predicts that the solvent shift of the harmonic should be twice that of the fundamental.

In conclusion, the first overtone of carbon monoxide has been observed in condensed phases of oxygen, nitrogen, and argon. Both the shape and half width are significantly changed in the transition of liquid solution to solid solution, while the band position is not appreciably altered in the phase change. (Changes were not observed for the methane-argon system [17].) The recent theory of Buckingham as well as the earlier theory ascribed to Kirkwood, Bauer, and Magat have been found not to apply to these systems. No explanation is apparent for the two overlapping bands observed for carbon monoxide in the clear crystalline argon solid. The explanation of Vu et al. [4] implies that a combination band involving the fundamental band and a lattice mode is more intense than the respective fundamental. This explanation is not consistent with the observation of one band for the vibration of methane in a clear crystalline argon solid [17]. The variation of the half width of the 0–2 band of CO in liquid oxygen in the temperature range of 57 to 82 °K cannot be readily explained with existing theories.

## Figures and Tables

**Table 1:** The 0–2 transition of CO in condensed oxygen, nitrogen and argon

Solvent	Phase	*T*	*v*	Δ*p*_½_
				
		*°K*	*cm^−1^*	*cm^−1^*
	gas	300	4260. 0	
O2	liq	57	4249.0 ±0.5	10.0 ±0.5
O2	liq	82	4249. 0 ±0. 5	18.4 ±0.5
N_2_	liq	78	4252. 4 ±0. 5	17.8 ±0.5
Ar	liq	82	4252.0 ±0.5	13. 7 ±0. 5
N_2_	solid	62	4252. 0 ±0. 5	12.3 ±0.5
Ar	solid	67	$\left[\begin{array}{c} 4245.0\pm 1.0\\ 4256.0\pm 1.0 \end{array}\right]$	25.0 ±2.0
